# Novel patient-derived tongue squamous cell carcinoma cell lines from non-smokers: 3D and in vivo models for drug response studies

**DOI:** 10.1007/s12032-026-03311-9

**Published:** 2026-06-29

**Authors:** Graziella Ribeiro de Sousa, Guilherme da Silva Carvalho, Bruna Miyoko Ikenaga de Brito, Gabriel da Silva, Maria Clara Rezende Barbosa Lopes, Pablo Shimaoka Chagas, Elvis Terci Valera, Graziela Vieira Cavalcanti, Luiz Carlos Conti de Freitas, Andréia Machado Leopoldino

**Affiliations:** 1 Department of Clinical Analyses, Toxicology and Food Sciences, School of Pharmaceutical Sciences of Ribeirão Preto, Bandeirantes Avenue, Ribeirão Preto, 14040-903 SP Brazil; 2 Department of Pediatrics, Ribeirão Preto Medical School, Ribeirão Preto Clinical Hospital, University of São Paulo, Ribeirão Preto, SP Brazil; 3 Department of Ophthalmology, Otolaryngology, Head and Neck Surgery, Ribeirão Preto Medical School, University of São Paulo, Ribeirão Preto, SP Brazil

**Keywords:** Tongue Squamous Cell Carcinoma (TSCC), Oral cancer cell line, Tumor spheroids, Drug response, Non-smoking patients

## Abstract

Tongue squamous cell carcinoma (TSCC) is the most prevalent and aggressive subtype of oral squamous cell carcinoma (OSCC), with a high incidence of lymph node metastasis even in early stages. The five-year overall survival rates remain low, around 50% in advanced stages. Cell line models are essential tools to understand the complexity of TSCC and develop new therapies. However, most commercially available TSCC cell lines are derived from patients with a history of tobacco use or have unknown exposure backgrounds. We established two novel TSCC cell lines, LMSCC03 and LMSCC16, derived from non-smoking and treatment naïve Brazilian patients. These cell lines were characterized by doubling time, 3D culture (spheroids and organoids), tumorigenicity through xenotransplantation in nude mice, immunohistochemical expression of key OSCC biomarkers and drug response. Genetic identity was confirmed by STR profiling. LMSCC03 and LMSCC16 displayed epithelial morphology and pan-cytokeratin staining. They demonstrated in vitro capacity to form spheroids and organoids, and generated tumors in vivo. STR profiling confirmed their novelty relative to existing cell lines in the DSMZ database. Protein analysis revealed high p53 nuclear levels in LMSCC16 cells. Interestingly, CD44 and c-Myc expression were observed only in fibroblast-enriched cultures, but not in the epithelial LMSCC03 and LMSCC16 cells. LMSCC16 also harbored two *TP53* mutations and showed increased resistance to Cisplatin and Paclitaxel compared to established TSCC cell lines. Cisplatin treatment in spheroids reduced *OCT4*, *CCND1*, *CCNB1*, and *CDH1* gene expression in LMSCC03. In contrast, LMSCC16 showed significant modulation of *OCT4*, increased *CCND1* and *CCNB1* expression, and reduced *CDH1* levels following treatment. We established two novel TSCC cell lines that represent clinically relevant models to explore TSCC carcinogenesis and therapeutic responses, particularly in non-smoking populations.

**Supplementary Information:**

The online version contains supplementary material available at 10.1007/s12032-026-03311-9.

## Introduction

Oral squamous cell carcinoma (OSCC) is the most prevalent type of head and neck cancer. According to the Global Cancer Observatory (GLOBOCAN), approximately 388,000 new cases were reported worldwide in 2022 [1]. OSCC develops from the mucosal epithelium of the oral cavity, with the tongue accounting for about 40% of all cases and diagnosed as tongue squamous cell carcinoma (TSCC) [2]. Smoking and alcohol consumption are the most common risk factors; however, a subset of TSCC arises in patients without these exposures, and only a small fraction of cases are associated with human papillomavirus (HPV) infection [3].

Most TSCCs exhibit a high incidence of regional lymph node (LN) metastasis since early stages [4]. The primary treatment for TSCC involves surgical intervention, such as glossectomy, often followed by adjuvant radiotherapy (RT) or chemoradiotherapy (CRT) in locally advanced cases to improve clinical outcomes [5]. Despite these interventions, recurrence rates are high, and the 5-year overall survival rate below 50% [6]. Furthermore, surgical resection of TSCC can significantly impact oral functions, including mastication, taste, and deglutition, leading to a poor quality of life [5]. These limitations underscore the need for improved biological models that better recapitulate TSCC behavior and support the development of more effective and personalized therapeutic strategies.

Human TSCC cell lines have been widely used in preclinical studies and have contributed to elucidating TSCC carcinogenesis [7]. To date, several tongue-derived cell lines have been established, and their clinical characteristics are summarized in Table S1. These cell lines were established from TSCC patients with a history of tobacco and/or alcohol use, or whose habits are unknown, and the lack of clinical annotation for many of them limits their translational value. Notably, only two TSCC cell lines from a never-smoking patient, UCSF-OT-1109 and UPCI-SCC-040, have been reported [8, 9], highlighting the need for more clinically defined models derived from treatment-naïve and etiologically diverse patients.

In the present study, we describe the establishment and characterization of two novel TSCC cell lines derived from treatment-naïve and non-smoking patients: LMSCC03 and LMSCC16. Both cell lines originated from pathological advanced stage, T3N3bM0, according to the American Joint Commission on Cancer Staging, 6th Ed. LMSCC16 harbored two distinct *TP53* mutations. These cell lines formed spheroids and organoids in vitro and tumors in nude mice, demonstrating self-renewal and tumorigenic capacity, with LMSCC03 exhibiting a higher rate than LMSCC16. Xenograft tumors from both cell lines retained key histopathological and molecular features of the original tumor biopsies. Overall, the data demonstrates that the applied methodology is efficient and robust for establishing cell cultures that grow as three-dimensional (3D) systems, enabling the consistent generation of LMSCC cell lines. These cell lines could be made available for others in the scientific community and represent a new model for further studies of TSCC biology, especially in the context of non-smoking and treatment-naïve patients.

## Methods and patients

### Patients and tumor tissues

Fresh tumor tissues from 27 OSCC were obtained from surgical resections performed at the Clinics Hospital from Ribeirão Preto Medical School (HC/FMRP-USP) with signed written informed consent. Clinicopathological data, including age, sex, anatomic site, histopathological stage, tumor differentiation, perineural invasion, vascular infiltration, treatment, alcohol intake, and smoking, were obtained and are presented in Table 1.

**Table 1:** Characteristics clinical and pathological features of OSCC patients (*n* = 27)

Characteristic (*n* = 27)	Total (%)
*Sex*	
Male	22 (81.5%)
Female	05 (18.5%)
*Age. years. median (range)*	61
*Anatomic site*	
Tongue	14 (51.9%)
Floor of the mouth	08 (29.6%)
Retromolar trigone	02 (7.4%)
Gingiva	02 (7.4%)
Tongue and floor of the mouth	01 (3.7%)
*Pathologic stage*	
I	01 (3.7%)
II	07 (25.9%)
III	06 (22.2%)
IV	13 (48.2%)
*Tumor differentiation*	
Well differentiated	03 (11.1%)
Moderately differentiated	17 (63.0%)
Poorly differentiated	05 (18.5%)
Unavailable data	02 (7.4%)
*Perineural invasion*	
Yes	16 (59.3%)
No	08 (29.6%)
Unknown	03 (11.1%)
*Vascular infiltration*	
Yes	10 (37.0%)
No	13 (48.2%)
Unavailable data	04 (14.8%)
*Alcohol Intake*	
Yes	22 (81.5%)
No	05 (18.5%)
*Smoking*	
Yes	22 (81.5%)
No	05 (18.5%)

## Primary cell culture

**Fig. 1:**
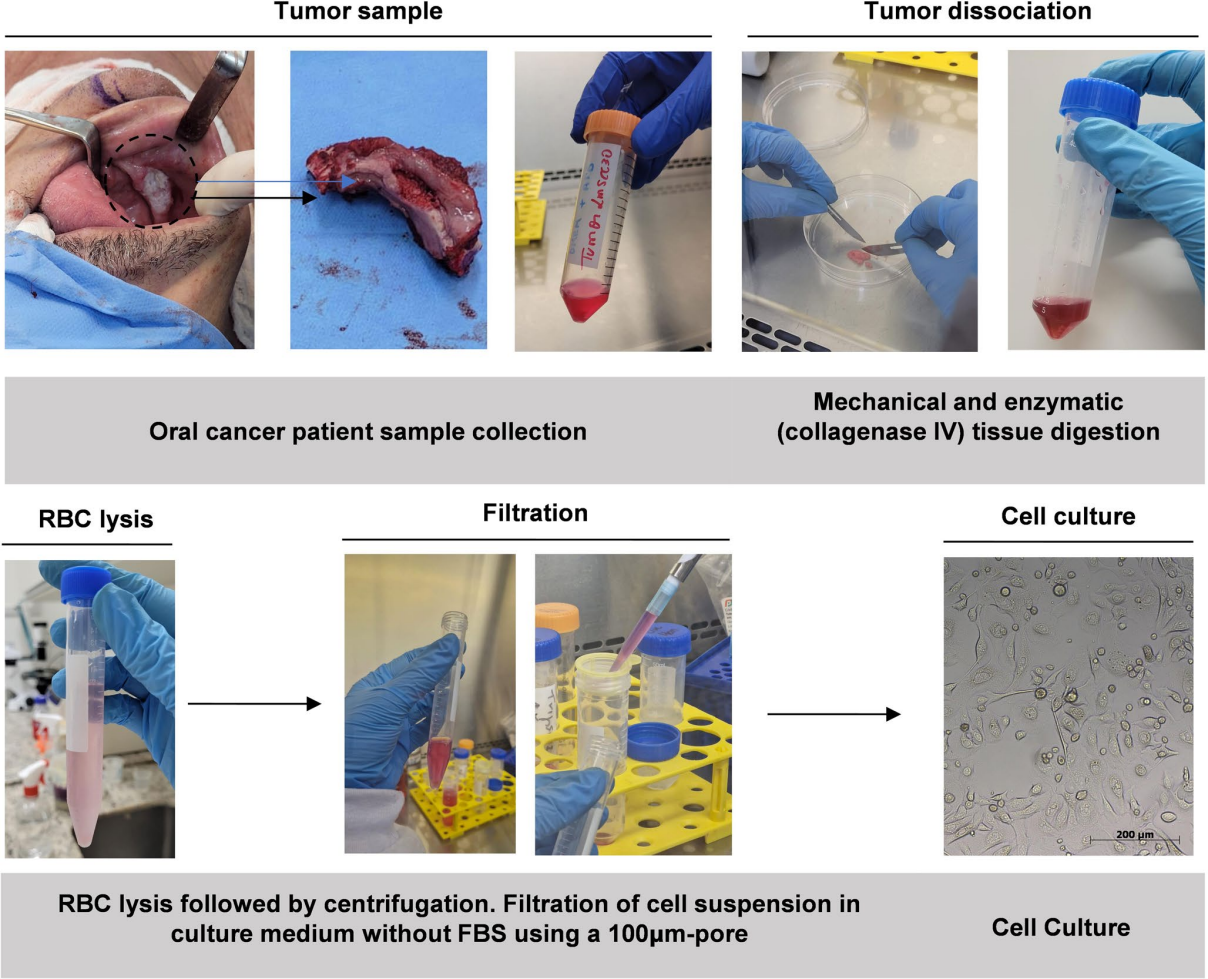
Workflow for the establishment of primary OSCC culture from surgical specimens. Schematic representation of the protocol is used to establish primary culture from OSCC fresh surgical specimens. *Step 1- Tumor sample collection*: A fresh tumor is collected immediately after surgical resection and stored in DMEM without fetal bovine serum (FBS). *Step 2- Tumor dissociation*: tissues are mechanically minced into ~ 0.2 cm^3^ fragments using a scalpel and enzymatically dissociated with collagenase IV for 60 min at 37 °C. *Step 3- Red blood cells (RBC) lysis*: the remaining cell suspension is treated with RBC lysis buffer to remove red blood cells. *Step 4-Filtration*: Cells are resuspended in DMEM and filtered through a 100 μm cell strainer. Viable cells are counted using Trypan Blue exclusion in a Neubauer chamber. *Step 5-Cell Culture*: Cells are plated in DMEM supplemented with 10% FBS, 1% penicillin/streptomycin, and maintained under standard culture conditions

Oral tumor tissues were fragmented into small pieces and enzymatically dissociated with collagenase IV (cat#17104019, Gibco). After digestion, red blood cells were lysed, and the cell suspension was filtered through a 100 μm strainer and cultured in Dulbecco’s Modified Eagle Medium (DMEM, Sigma-Aldrich) supplemented with 10% fetal bovine serum (FBS, Gibco), 1% Penicillin/Streptomycin (Hyclone, #SV30079.01). The step-by-step process is illustrated in Fig. 1. Methodological details are provided in the Supplementary Material.

## Short tandem repeat (STR) profiling

Genomic DNA was extracted using PureLink™ Genomic DNA Mini Kit (Invitrogen) from the LMSCC03 (passage #20 and #40) and LMSCC16 (passage #10 and #12). Sixteen STR loci were amplified by polymerase chain reaction (PCR) and compared with the profiles in DSMZ for reference (https://www.dsmz.de).

## Western blot

Proteins from cells at different passages were separated by SDS-PAGE and transferred to membranes as described [10]. Primary antibodies were used for p53 (#2527), c-Myc (#5605), CD44 (#3570S), GAPDH (#2118) from Cell Signaling Technology, PCK26 (Abcam, ab6401), α-SMA (SAB2500953, Sigma-Aldrich) and Vimentin (sc-3232, Santa Cruz Biotechnology). Immunocomplex intensities were quantified using ImageJ software. Additional details are found in the Supplementary Material.

### *TP53* DNA sequencing

The *TP53* gene was amplified from genomic DNA using GoTaq^®^ G2 Flexi DNA Polymerase (Promega) as described by [11]. PCR products were sequenced using BigDye Terminator v1.1 Cycle Sequencing Kit (Applied Biosystems) on an ABI3130xL sequencer. Sequences were aligned to the human genome (GRCh38) using the Codon Code Aligner software.

## Doubling time and spheroids formation assay

LMSCC cells were seeded in 96-well plates, and viability was assessed at 24, 48, 72, and 96 h using the MTT assay. For spheroid formation, LMSCC03 and LMSCC016 cells were cultured in 24-well plates pre-coated with 3.0% Poly-HEMA polymer (Sigma-Aldrich, #P3239) and monitored for 7 days. The spheroid’s size was measured using ImageJ.

### Matrigel-embedded oganoids formation

For matrigel-embedded organoids from patients LMSCC03 and 16 cells, a mixture of DMEM-F12 Advanced (AdDMEM/F12, cat # 12634–034, Life Technologies) was supplemented with 1mM HEPES, 10 μm Y27632 (ROCK inhibitor) (Caynam #10005583), 50ng/mL EGF (Peprotech, #AF-100-15-500ug), 50ng/mL bFGF (Thermo Fisher, #PHG0368), 1% Pen/Strep (Hyclone, #SV30079.01), L-Glutamine 1X (Sigma-Aldrich, #G7513), 100ug/mL de Primocin (InvivoGen, #PML-45-11) and embedding in 3:4 of Matrigel (Corning, cod. CLS354234). Plates were incubated for 30 min at 37 °C and 5% CO_2_ to solidify the mixture before addition of 100 uL of DMEM-F12 Advanced supplemented as previously described by [12].

### Cell viability in 2D cultures and organoids, and cell cycle assay

LMSCC cells for 2D assay were seeded in 96-well plates and treated with Cisplatin (10, 20, 50, 100µM) or Paclitaxel (1, 5, 10, 20nM) for 72 h. Viability was measured by MTT assay. Organoids were cultivated as described above and were treated with Cisplatin (5, 10, 20µM) and analyzed with CellTiter-Glo^®^ 3D (Promega, #G9683). For cell cycle analysis, cells were synchronized and harvested at 24 and 48 h as described by [10]. Detailed information about organoids viability and cell cycle protocols is found in the Supplementary Material.

## Quantitative Reverse Transcription PCR (qRT-PCR)

Relative and absolute mRNA expression levels were measured by quantitative real-time PCR using SYBRGreen (PCR Biosystems, USA) to evaluate *OCT4*, *SOX2*, *NANOG*, *CCND1*, *CCNB1*, *CDH1*, and *CDH2* after control and Cisplatin treatment (IC₅₀ previously established-LMSCC16: 45µM and LMCC03:18µM). Cells were cultured as spheroids in pre-coated plates with 3.0% Poly-HEMA polymer for 48 h. Primer sequences are listed in Supplementary Table S4. The reactions were performed on Mastercycler^®^ ep realplex (Eppendorf) using *RPL27* as an endogenous [13]. The data were analyzed using the 2^−ΔΔCT^ method for relative expression compared to the control group (untreated), while absolute expression between LMSCC03 and LMSCC16 was determined based on 10,000/2^ΔCT^ [14].

### In vivo tumorigenic analysis

Animal experiments were approved by the Animal Ethics Committee of the University of São Paulo, Ribeirão Preto (CEUA FCFRP nº 24.1.145.60.8). LMSCC03 (1.2 × 10^6^) and LMSCC016 cells (2.5 × 10^6^) were resuspended in a 1:1 Matrigel/DMEM medium and subcutaneously transplanted into the flanks of BALB/c nude mice. LMSCC03 were transplanted into three mice (six injections in total), whereas two mice received LMSCC16 (four injections in total). Animals were monitored daily, and tumor growth was measured 2–3 times per week using a digital caliper. Tumor volume was calculated as *(length × width²)/2*, where length represents the longest tumor diameter and width the perpendicular diameter. At the endpoint, mice were humanely euthanized, and tumors were processed for histopathological and immunohistochemical analyses as detailed in the Supplementary Material.

## Immunohistochemistry

Immunohistochemistry (IHC) was performed on formalin-fixed paraffin-embedded (FFPE) xenograft (LMSCC03 and LMSCC16) tumors and paired patient tissue sections using a polymer-based detection system (Two Step Polymer Immunohistoprobe Plus kit, Advanced Biosystems), as described in the Supplementary Material. Proteins were analyzed using antibodies against Ki67 (M7240, Dako), CD44 (#3570), E-cadherin (#3195), both from Cell Signaling Technology, PCK26 (Abcam, ab6401), and p53 (sc-126, Santa Cruz Biotechnology). Images were acquired at ×20 magnification using an Aperio ScanScope Scanner (Aperio Technologies).

### Statistical analysis

Statistical analyses were performed using GraphPad Prism 11.0. Comparisons between the two groups were performed using an unpaired Student’s t-test, and among multiple groups using a One-way ANOVA test, followed by Tukey’s or Sidak’s post-test. Functional assays are reported as mean ± SD of three independent experiments, performed in duplicate or triplicate. WB and qRT-PCR experiments were independently replicated at least twice. *p-value* < 0.05 was statistically significant.

## Results

### Clinicopathological features of the OSCC patient cohort

A total of 27 OSCC tissue samples were collected surgically from patients, with clinical data summarized in Table 1. Most OSCC patients aged 50–69 (25/27, 92.6%). Higher prevalence occurred among men (22/27, 81.5%). Anatomically, the tongue was the most common site for OSCC, representing 51.9% (14/27), followed by the floor of the mouth (8/27, 29.6%), retromolar trigone (2/27, 7.4%), and gingiva (2/27, 7.4%). Notably, one patient presented with simultaneous tumors in both the tongue and floor of the mouth at diagnosis. The pathological stage revealed a predominance of advanced tumors, with 70.4% (19/27) classified as T3 and T4. Only a single patient had a T1 tumor. Perineural invasion was observed in 59.3% (16/27) of tumors, while vascular infiltration was found at 37.0% (10/27). These pathological features underscore the advanced and invasive nature of OSCC in this cohort. Most patients had a history of smoking and alcohol consumption (22/27, 81.5%). None of the patients had received any treatment before surgical resection. After resection, RT was the most frequently used treatment (15/27, 55.6%), followed by CRT (8/27, 29.6%). Nine patients experienced tumor relapses (< 6 months), and twelve patients died (< 15 months).

### Establishment and characterization of human primary OSCC cell cultures

Fresh OSCC tumor fragments were processed and cultured as shown in Fig. 1. Of 27 tissues, 15 (55.6%) successfully grew in vitro. Six cultures (22.2%) failed to establish growth, and one (3.7%) yielded insufficient viable cell post-processing. Five cultures (18.5%) were lost due to bacterial and/or fungal contamination. Among the established cultures, 46.7% (7/15) exhibited typical fibroblast morphology (spindle shapes) in more than 90% of cells and were classified as “fibroblast-like”. In contrast, 53.3% (8/15) formed clusters with epithelial (round or polygonal) and fibroblast-like cells, designated as mixed-type culture. Protein analysis showed alpha-smooth muscle actin (alpha-SMA), a cancer-associated fibroblast (CAF) marker, in 64.3% (9/14) of cultures, with a heterogeneous pattern of vimentin and pan-cytokeratin, as epithelial markers, across samples (Figures S1A-B, Table S2).

**Fig. 2:**
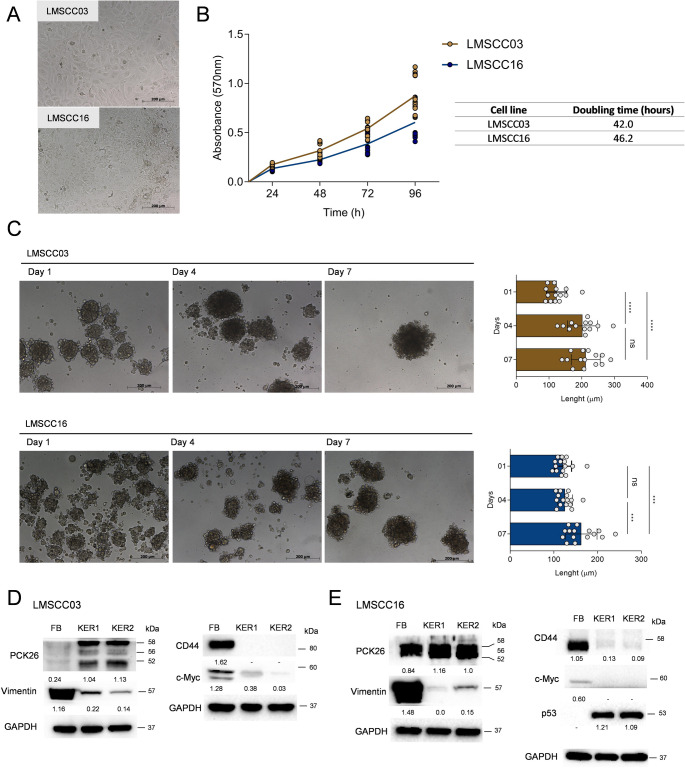
LMSCC03 and LMSCC16 cell morphology, doubling time, spheroids formation, and protein markers profile. (**A**) Representative phase-contrast images showing the morphology of LMSCC03 and LMSCC16 cells. Images were captured using a 20x objective; scale bar: 200 μm. (**B**) Doubling times analysis based on the cell growth of LMSCC03 was 42.0 h and LMSCC16 = 46.2 h. Data from three independent experiments, each performed with 4–6 replicates, are presented as mean ± SD. (**C**) Spheroids generated with LMSCC03 and LMSCC16 cells cultured in non-adherent culture microplates and analyzed on days 1, 4 and 7 using an inverted microscope (Zeiss Axiovert 40); images were taken with 20x objective. Bar in each panel: 200 μm. The graph displays spheroid diameter (µm). Statistical significance: *p < 0.05, **p < 0.001, ***p < 0.0001. (**D**) Western blot analysis of pan-cytokeratin (PCK26), vimentin, c-Myc, CD44, and p53 proteins in mixed-cells (fibroblasts and epithelial cells, designed as FB due to presence of fibroblasts-like cells) and purified epithelial cells (KER) populations from LMSCC03 at passages #10 (KER1) and #11 (KER2), and (**E**) LMSCC16 at passages #8 (KER1) and #10 (KER2). GAPDH was used as a loading control and the expression calculated using ImageJ software

The mixed-type cells were maintained in culture, undergoing passage when they reached high confluence, and as a result, two new cell lines of TSCC were established, denoted as LMSCC03 and LMSCC16. LMSCC03 originated from an 84-year-old female non-smoking and non-alcoholic drinker diagnosed with a tongue squamous cell carcinoma staged as pT3N3b. The patient died five months after initial diagnosis without any treatment. LMSCC16 was derived from a 77-year-old male non-smoker with daily alcohol intake and a tongue tumor staged as pT3N3b. This patient was treated with full-dose radiotherapy without concomitant chemotherapy and died six months after diagnosis. Pure epithelial cell cultures were established after approximately eight (LMSCC03) and six months (LMSCC16) using selective trypsinization to eliminate contaminating fibroblasts. These cultures exhibited stable growth rates and consistent epithelial morphology with positive pan-cytokeratin, being designated as KER (Fig. 2A).

### LMSCC03 and LMSCC16 demonstrate stable proliferation and 3D growth capacity

LMSCC03 and LMSCC16 proliferated stably as adherent monolayers for more than 40 (∼23 months) and 36 passages (∼17 months), respectively. Their doubling times were 42 h (LMSCC03) and 46.2 h (LMSCC16) (Fig. 2B). Cell cycle analysis corroborated these results, showing a faster progression through the cell cycle for LMSCC03 compared to LMSCC16 (Figures S2A-B). Both cell lines formed spheroids in Poly-HEMA pre-coated plates within 7 days (Figs. 2C-D). However, the spheroids of LMSCC16 cells were significantly smaller than those of LMSCC03. In addition, both cells grew as three-dimensional (3D) organoids in Matrigel-embedded cultures (Figure S2C).

Protein analyses of LMSCC03 and LMSCC16 cells revealed a profile consistent with fibroblast-like and epithelial (KER) cells. Pan-cytokeratin was significantly reduced in FB cells compared to the epithelial cells, while vimentin was higher in the FB population (Figs. 2D-E). Interestingly, CD44 and c-Myc were detected in FB cells but were absent in the epithelial cells (KER1 and KER2). Conversely, p53 protein levels were higher levels in epithelial cultures compared to FB ones, particularly in LMSCC16, whereas it was undetectable in LMSCC03 (Figs. 2D-E). STR profiling at 16 *loci* confirmed the individual profiles of these new TSCC-established cell lines, with no matches found in the DSMZ cell line database (Table S3).

### LMSCC cells exhibit tumorigenicity in the xenograft model

**Fig. 3:**
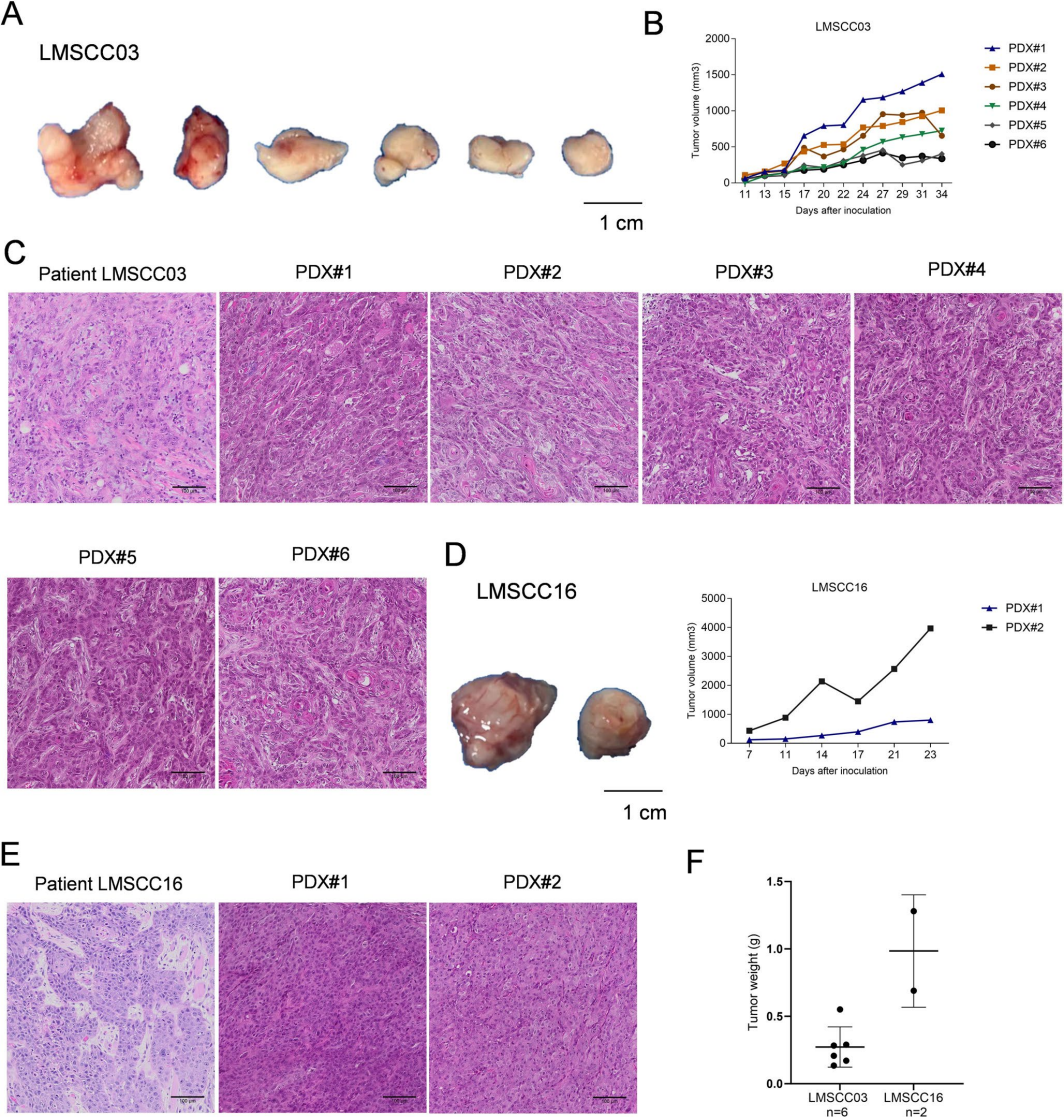
LMSCC03 and LMSCC16 xenograft tumor models recapitulate patient tumor histopathology. (**A**) Representative images showing six xenograft tumors generated by subcutaneous transplantation of the LMSCC03 cell line into the right and left flanks of BALB/c nude mice. (**B**) Tumor volumes were measured weekly for 34 days. The graph represents the volume of xenografts LMSCC03 tumors. (**C**) Hematoxylin and eosin (H&E) staining of slices of LMSCC03 tumor and its corresponding patient tumor tissue, demonstrating histopathological features. (**D**) Images of xenograft tumors generated with LMSCC16 cells transplanted into the flanks of BALB/c nude mice, with tumor volume monitored over 23 days. (**E**) H&E staining of slices of LMSCC16 tumor and its corresponding patient tumor tissues. Original magnification: 200x; scale bar:50 μm. (**F**) Comparison of tumor weights at the end of the experiment for xenograft LMSCC03 and LMSCC16 tumors, showing a larger tumor mass formed by LMSCC16 cells

The tumorigenicity of LMSCC03 was evaluated by subcutaneous transplantation into the flanks of three BALB/c nude mice (six injections in total) (Fig. 3A). All LMSCC03 xenografts (6/6) developed tumors within 12 days, resulting in a 100% tumor take rate (Fig. 3B). In contrast, LMSCC16 exhibited lower tumorigenicity, with a 50% tumor take rate (2/4) at 23 days (Fig. 3D). The reduced number of LMSCC16 transplantations reflects the low cell number at that time. No macroscopic metastases were detected in the liver or lungs upon necropsy. Histology analyses showed that tumors resembled the corresponding primary tumors, characterized by large, hyperchromatic nuclei and increased mitotic figures, with similar features observed within the same PDXs. Notably, the two LMSCC16-derived xenografts displayed increased intratumoral fluid content, suggestive of an inflammatory component (Fig. 3C and E). Tumor growth curves and volume measurements are shown in Fig. 3B and D. LMSCC03 exhibited higher mRNA levels of genes associated with stemness (*OCT4* and *NANOG*), cell cycle regulator (*CCNB1*), and cell-cell adhesion (*CDH1*, E-cadherin) compared with LMSCC16. (Figure S3). In contrast, *CDH2*, which encodes N-cadherin, was significantly higher in LMSCC16 than in LMSCC03. No significant differences in *SOX2* and *CCND1* expression genes were observed between the two cell lines (Figure S3). These findings suggest that LMSCC03 exhibits enhanced proliferative capacity and tumorigenicity compared to LMSCC16. Nevertheless, both TSCC models retain histopathological features consistent with primary tumors (Fig. 3A).

### Molecular characterization reveals *TP53* mutation and epithelial marker staining

**Fig. 4:**
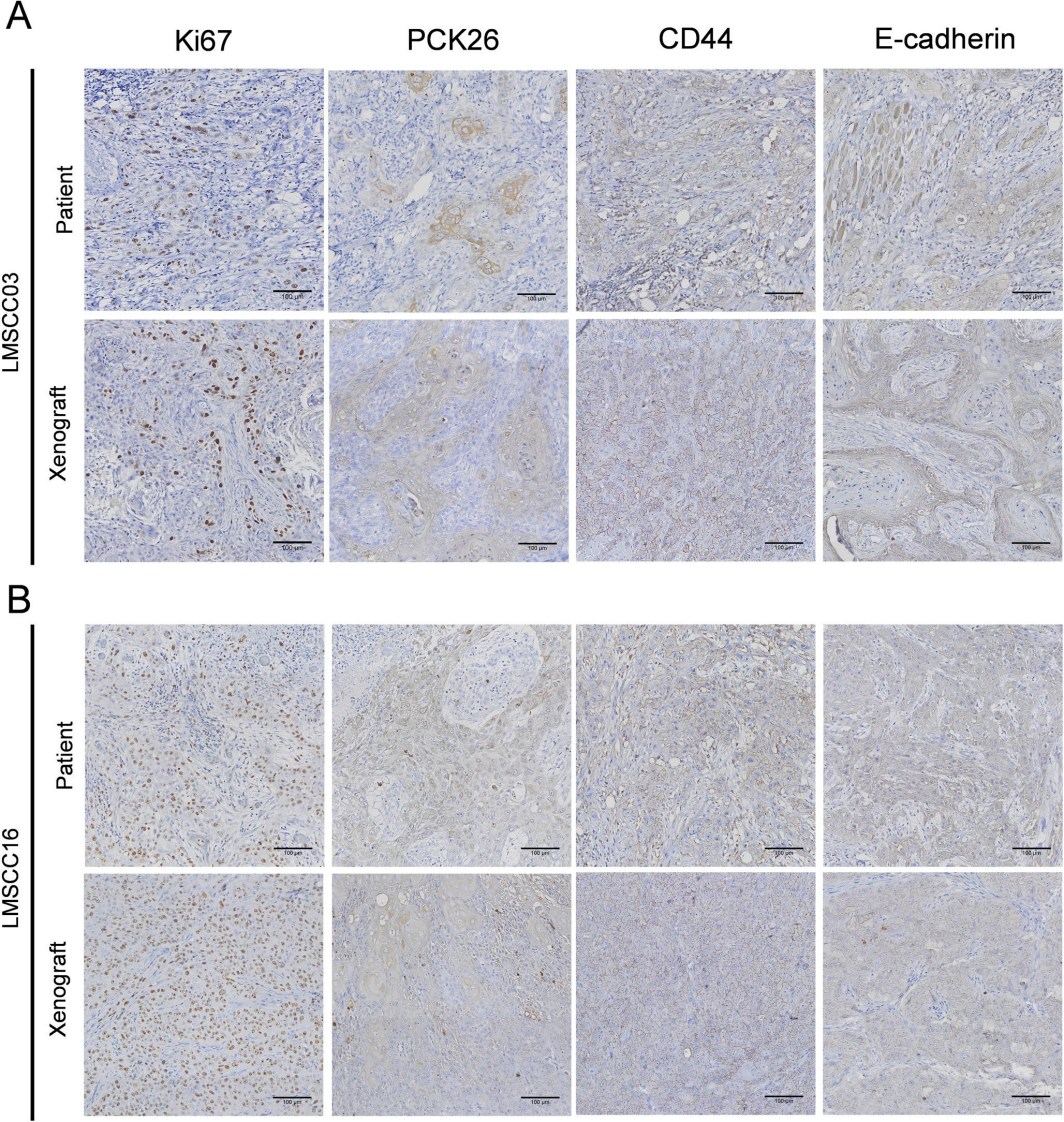
Immunostaining of xenograft LMSCC03 and LMSCC16 tumors and their corresponding patient TSCC tumors. (**A**) Representative immunohistochemical (IHC) staining of patient tumor tissue (top) and LMSCC03-derived xenograft tumor (bottom) for Ki-67, pan-cytokeratin (PCK26), CD44, and E-cadherin. (**B**) IHC staining of patient tumor (top) and LMSCC16-derived xenograft tumor (bottom) for the same markers. Images were acquired at original magnification: 200x; scale bar: 50 μm

**Fig. 5:**
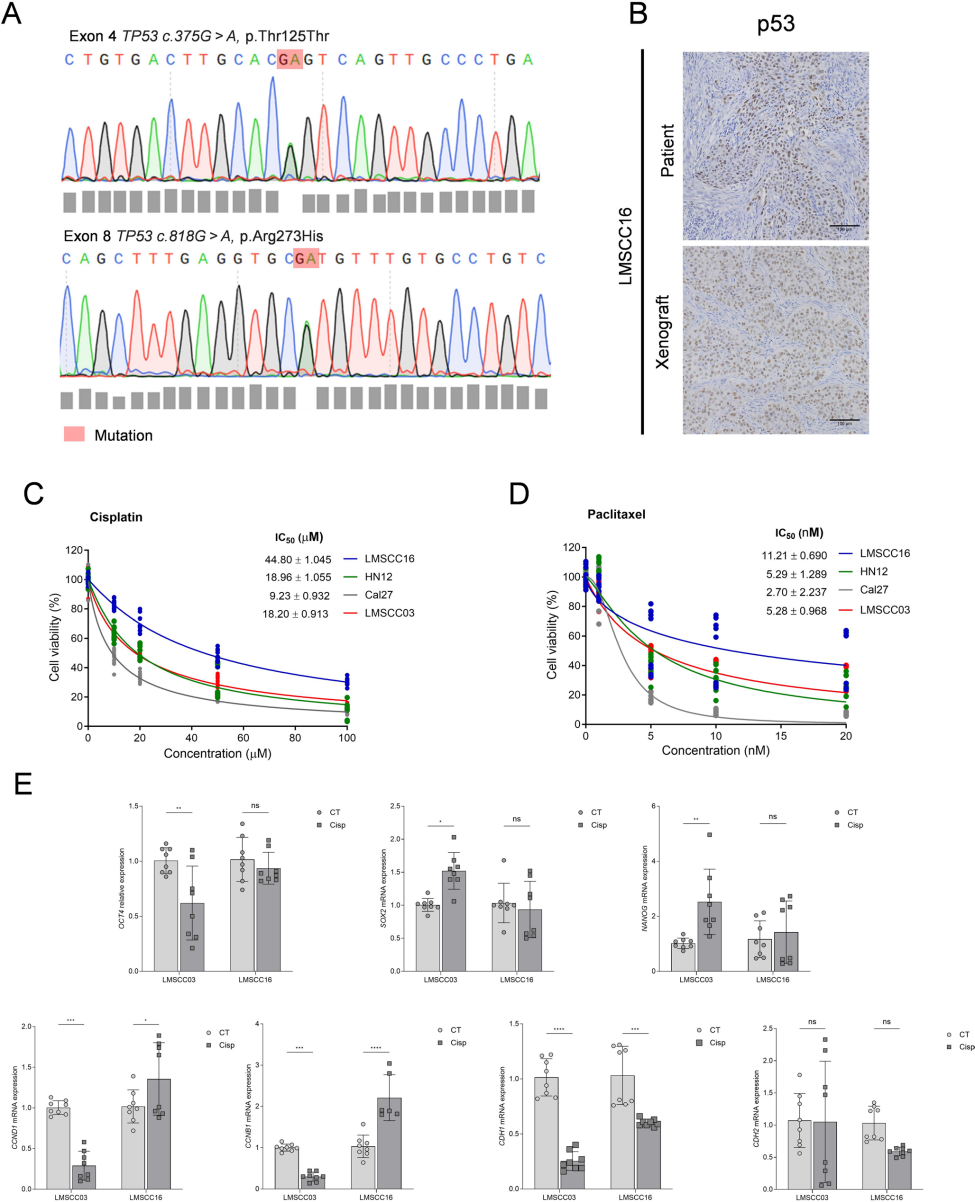
*TP53* mutation analysis and drug sensitivity in LMSCC03 and LMSCC16 cells. (**A**) Sanger sequencing validation of two somatic *TP53* mutations identified in LMSCC16 cells: a synonymous mutation in exon 4 (c.375G > A, p.Thr125Thr) and a missense mutation in exon 8 (c.818G > A, p.Arg273His). (**B**) Representative IHC staining of p53 protein showing its nuclear accumulation in both LMSCC16-derived xenograft tumor and corresponding patient tumor tissue. (**C**) Dose-response curves of LMSCC03, LMSCC16, and two known TSCC cell lines (Cal27 and HN12) treated with increasing concentrations of Cisplatin and (**D**) Paclitaxel for 72 h. Cell viability was assessed by MTT assay. The half-maximal inhibitory concentration (IC₅₀) values were calculated using log non-linear regression in GraphPad Prism. (**E**) Relative mRNA expression using RT-qPCR of pluripotency markers (*OCT4*, *SOX2*, *NANOG*), cell cycle regulators (*CCND*1, *CCNB1*), and epithelial–mesenchymal transition markers (*CDH1* and C*DH2*) in LMSCC03 and LMSCC16 cells. Cells were treated with Cisplatin (LMSCC16: 45µM and LMCC03: 18µM) for 48 h and compared to untreated control cells. Statistical analysis was performed using Two-Way ANOVA followed by Sidak’s post hoc test. Significance levels were defined as ns > 0.05; *p* < 0.05; **p < 0.01; *p < 0.001; ***p < 0.0001

Immunostaining confirmed the high level of Ki67, a proliferation marker, in both patient TSCC tissues and xenograft tumors formed by LMSCC03 and LMSCC16 cell lines. Markers such as PCK26, CD44, and E-cadherin were positively stained, supporting the epithelial origin with a similar pattern in both tissues (Figs. 4A-B). DNA sequencing analysis of the full *TP53* gene revealed LMSCC03 as a *wild-type TP53* sequence. In contrast, LMSCC16 cell harbored two heterozygous mutations: exon 4 (codon 375, c.375G > A) and exon 8 (codon 818, c.818G > A) (Fig. 5A). Immunohistochemical analysis showed a substantial nuclear accumulation of p53 protein in LMSCC16 tumor tissue derived from the patient and the corresponding xenograft tumor (Fig. 5B).

### Distinct cisplatin sensitivity profiles in LMSCC03 and LMSCC16 cells

Cisplatin and Paclitaxel are commonly used chemotherapeutic agents for OSCC. Drug sensitivity assays showed that LMSCC03 exhibited IC_50_ values of 18.20 µM for Cisplatin (Cis) (Fig. 5C) and 5.28 nM for Paclitaxel (Fig. 5D). In contrast, LMSCC16 was more resistant compared to LMSCC03 and other TSCC cell lines (Cal27 and HN12), with IC_50_ of 44.80µM for Cisplatin (Fig. 5C) and 11.21 nM for Paclitaxel (Fig. 5D). Additionally, organoids derived from both LMSCC cell lines confirmed sensitivity to Cisplatin, with a significant reduction in organoid size and cell viability (Figure S2D). To further assess Cisplatin-induced molecular changes, mRNA levels of pluripotency, cell cycle, and EMT markers were evaluated in control and treated groups. In LMSCC03, Cis reduced the expression of *OCT4*, *CCND1*, *CCNB1*, and *CDH1*, while increasing *SOX2* and *NANOG*. No significant changes were observed in *CDH2* expression after treatment (Fig. 5E). In a distinct manner, LMSCC16 showed upregulation of *CCND1* and *CCNB1*, accompanied by *CDH1* reduction. *CDH2* expression remained unchanged in both cells (Fig. 5E).

## Discussion

 In vitro cell-based and animal models have proven essential tools for advancing our understanding of OSCC biology, including tumor heterogeneity and therapeutic resistance mechanisms, which have been associated with poor clinical outcomes [15]. However, it is known that the efficiency of establishing cell lines directly from oral tumor tissues is low [7]. In our study, we achieved a 55.5% success rate in cultivating OSCC primary tumor cells using mechanical disaggregation and collagenase digestion of the tumor tissues. In comparison, White et al. [9] successfully developed head neck squamous cell carcinoma (HNSCC) cell lines from 26.1% of 199 samples, with many cultures producing only fibroblasts (51.8%) or failing due to contamination (9.5%). Heo et al. similarly reported a 24% success rate [16]. These reports highlight the critical technical challenges of primary OSCC culture [16]. The use of Primocin™ (InvivoGen, 100 µg/mL) in our culture protocols was a key factor in reducing contamination and for cell growth success.

Fibroblast overgrowth [17, 18] has been related to the high proportion of CAFs, which can reach 80% of the HNSCC mass in advanced stages [19]. Consistent with this, all primary OSCC cultures showed fibroblast growth, and eight cultures demonstrated epithelial cells. Using differential trypsinization, as described by White et al. [9] and Gawas et al. [7], we selectively depleted fibroblast cells and established two pure epithelial tumor cultures, which have been maintained for at least 30 passages.

Demographically, over 80% of patients from our cohort were male, and 85% were > 50 years old, in line with OSCC epidemiology [2]. In our study, 83.3% of female patients were over 60 years old, compared to 42% of male patients. Similarly, Kruse et al. [20] and Lin et al. [21] reported a higher prevalence of OSCC among older females (> 65 years) than men, suggesting age- and sex-specific risk factors for females. While the number of females in our cohort is limited, this finding warrants further investigation.

The tumors obtained from the tongue are the most prevalent (51.8%), aligning with other studies that described a high incidence of TSCC in OSCC [9, 22, 23]. This may be due to the tongue’s rich vascularity, epithelial turnover, direct exposure to carcinogens, and chronic inflammation due to repeated friction and trauma [23]. Here, we reported the establishment of two primary cell lines from treatment-naive TSCC patients: LMSCC03 and LMSCC16. LMSCC03 was derived from an 84-year-old female, a non-smoker and a non-alcoholic, and LMSCC16 was from a 77-year-old male patient, a non-smoker with moderate alcohol consumption. At the same time, tobacco and alcohol remain key TSCC risk factors, with more than 85% of patients smoking, drinking, or consuming betel nut [24]. Recent studies have shown a rising incidence in young, non-smoking, and non-drinking females, potentially linked to HPV infection, immune dysfunction, and genetic origin (Li-Fraumeni syndrome, Fanconi’s anemia) [25]. Although the HPV status in LMSCC16 is unknown, most of the TSCC patients, including never-smokers, are HPV-negative [3]. TSCC cell lines from non-smokers are rare. Recently, Wang et al. reported the UCSF-OT-1109 cell line as an experimental model without background smoke [8]. LMSCC16 and LMSCC03 offer valuable models for exploring molecular mechanisms beyond tobacco and alcohol.

We characterized the two TSCC cell lines by doubling time, spheroid and matrigel-embedded organoid formation, cell stemness markers, tumorigenicity in nude mice, and drug sensitivity. LMSCC03 and LMSCC16 exhibited relatively longer doubling times than some OSCC lines [7]. Both LMSCC cell lines formed spheroids in suspension culture, consistent with the study by Gawas et al. They reported spheroid formation in three primary cell lines (ACOSC3, ACOSC4, and ACOSC1) with a high level of cancer stem cell (CSC) markers, such as CD44 [7]. In our study, early-passage cells (mixed cells with fibroblasts) expressed higher levels of c-Myc and CD44 than the established pure epithelial cell lines, reinforcing the evidence that the presence of CAF contributes to the stemness profile. According to Zhang et al., high levels of lactic acid derived from CAFs promote a CSC-like phenotype in OSCC [26]. In vivo, TSCC cells formed moderate and poorly differentiated tumors, recapitulating key epithelial tumor markers, such as pan-cytokeratin, CD44, ki-67, and E-cadherin.

Interestingly, CD44 was detected in xenograft tumors from LMSCC03 and LMSCC16 cells. Of note, CD44 expression is strongly influenced by the tumor microenvironment and can vary substantially in vitro and in vivo conditions. Microenvironment cues, such as cytokine signaling, hypoxia, extracellular matrix interactions, and immune cell contact, have been shown to sustain CD44 expression in vivo [27]. Thus, the low CD44 detection in pure epithelial cultures in vitro and its presence in xenograft tumors likely reflect microenvironmental effects, highlighting the limitations of simplified in vitro models in recapitulating tumor complexity. 

*TP53* mutations affect approximately 80% of TSCC cell lines with available status and are associated with poor overall survival [28]. LMSCC16 harbors two *TP53* mutations, including a well characterized gain-function variant (p.R273H), previously reported in colorectal [29] and breast cancers [30] but not previously described in TSCC samples or cell lines. This lineage exhibited nuclear p53 accumulation and increased drug resistance. Although our data are correlative and do not establish causality, the presence of the p.R273H variant, together with nuclear p53 accumulation and a drug-resistant phenotype, supports the value of LMSCC16 as a relevant model to explore the functional impact of *TP53* mutations in TSCC in future mechanistic studies and the development of new therapeutics.

Cisplatin response was markedly heterogeneous between the two cell lines. LMSCC03 cell line exhibited reduced *OCT4* expression after treatment, suggesting sensitivity to cisplatin [31]. However, the increased gene expression of *SOX2* and *NANOG* indicates that a cellular subpopulation may have been selected during treatment, possibly activating escape mechanisms and acquiring a more resistant phenotype previously described in other tumors, such as bladder cancer [32]. In contrast, LMSCC16 maintained the expression of these genes even after treatment. This behavior, together with the IC_50_ values, suggests an intrinsically more cisplatin-resistant profile [33].

Cisplatin exposure exerts its antitumor effects primarily through the induction of DNA damage and subsequent activation of cell cycle checkpoints [34]. LMSCC03 demonstrated significant downregulation of *CCND1* and *CCNB1* gene expression, indicating effective checkpoint activation in response to DNA damage. Conversely, the LMSCC16 cell line showed increased expression of *CCND1* and *CCNB1*, suggesting a potential escape from checkpoint control mechanisms or an enhanced ability to tolerate DNA damage [35]. The maintenance or upregulation of these cyclins under genotoxic stress is frequently associated with therapeutic resistance, as it allows the preservation of proliferative signaling despite cellular damage [36].

When comparing both cell lines, additional differences were observed in *CDH1* and *CDH2* expression profiles. LMSCC03 exhibited higher *CDH1* and lower *CDH2* expressions, indicating a more epithelial phenotype. In contrast, LMSCC16 displayed an opposite pattern, consistent with mesenchymal features, which are often associated with increased cellular plasticity and therapeutic resistance. Collectively, the findings on cyclin D1 and cyclin B1 levels, together with the cell adhesion profile, are consistent with the literature and suggest that LMSCC03 is more sensitive to cisplatin treatment, whereas LMSCC16 presents an intrinsically resistant phenotype. In conclusion, our findings demonstrate the feasibility and relevance of establishing new patient-derived TSCC cell lines. These models, including 3D spheroid and matrigel-embedded organoid systems as well as in vivo, provide a translationally relevant framework to investigate tumor heterogeneity, therapeutic resistance, and personalized drug responses in TSCC from non-smokers patients.

## Supplementary Information

Below is the link to the electronic supplementary material.

Supplementary Material 1

Supplementary Material 2

Supplementary Material 3

Supplementary Material 4

Supplementary Material 5

## Data Availability

No datasets were generated or analysed during the current study.
