# Supplementary material for: Novel patient-derived tongue squamous cell carcinoma cell lines from non-smokers: 3D and in vivo models for drug response studies
Source: Med Oncol. 2026 Jun 29;43(8):206. doi: 10.1007/s12032-026-03311-9 (PMC13314703; doi:10.1007/s12032-026-03311-9)
Supplement: Supplementary file 1 — Supplementary Material 1 [file 12032_2026_3311_MOESM1_ESM.docx]

***Supplementary Material***

**Methods**

**Primary cell culture**

Oral tumor tissues were received within 30-60 minutes after surgical resection and the tissues were fragmented into small pieces with the assistance of a scalpel blade. Initially, the tissue pieces were dissociated with collagenase IV for 60 min at 37ºC. After complete digestion, the cells were centrifuged and resuspended in red blood cell lysis buffer. Next, the cells were centrifuged, resuspended in Dulbecco's Modified Eagle Medium (DMEM, Sigma-Aldrich) supplemented with 10% fetal bovine serum- FBS (Gibco), 1% Penicillin/Streptomycin (Hyclone, #SV30079.01), and filtered through a 100μm cell strainer. The filtered cells were counted as the number of viable cells using Trypan Blue. The cultures were maintained in a humidified 5% CO_2_ atmosphere at 37 °C.

**Epithelial cells isolation**

For epithelial-like cells isolation, primary tumor cells were subjected to a two-step selective trypsinization. In the first step of the selective trypsinization, cells were incubated with 0.25% trypsin-EDTA (T4049, Sigma-Aldrich) for 1 minute at 37ºC . The supernatant containing detached cells was immediately collected and neutralized with twice the volume of DMEM 10%FBS, to obtain the fibroblast-enriched fraction. Subsequently, fresh trypsin-EDTA solution was added to the remaining adherent cells and incubated for an additional 3-4 minutes at 37ºC. After gentle pipetting (up-a-down), the solution was neutralized with DMEM 10% FBS, and resulting cell suspension was collected to isolate the epithelial-like cell population. In each round, fibroblast-enriched cells were detached and collected for subsequent culture, while the remaining cells were retained in culture. This process was repeated multiple times to selectively enrich epithelial-like cells and minimize contamination from non- epithelial cells. The cultures were maintained in a humidified 5% CO_2_ atmosphere at 37 °C.

**Organoids cell viability**

Organoids were cultivated for two days before treatment with Cisplatin. After 72 hours of treatment, viability was assessed by disaggregating the organoids using dispase (Sigma-Aldrich, 5 mg/mL), followed by shaking the plate for 5 minutes at 800 rpm, then adding the CellTiter-Glo® 3D reagent (Promega) to a white well plate. Viable cells were measured using a SpectraMax ID3 microplate reader, and results were expressed as the percentage of viable cells relative to the untreated control. Organoids were observed by bright field microscopy using an inverted microscope (Zeiss Axiovert 40).

### **Doubling time assay**

Cells were incubated for 2 hours with media containing MTT (500 µg/mL), which is converted to formazan salt by mitochondrial enzymes. Next, formazan was solubilized with DMSO, and the absorbance reading was performed in a Synergy 2 plate reader at 570 nm (BioTek). The values of absorbance at each time point were plotted and the doubling time was calculated using the online tool https://www.omnicalculator.com/biology/cell-doubling-time. The assays were performed in independent biological triplicate, and the data were presented as mean ± standard deviation.

### **Western blot**

Cells at different passages were collected in lysis buffer (Sigma-Aldrich) and sonicated. Protein samples (~20 µg) were separated by SDS-PAGE and transferred onto 0.45µm PVDF membranes (GE Healthcare). Immunoreactive proteins were detected using SuperSignal West Dura Substrate (Thermo Fisher Scientific) and imaged on a ChemiDoc MP system (Bio-Rad).

**Cell cycle assay**

LMSCC03 and LMSCC016 were synchronized at G2/M phase by cultivation in medium containing 0.25 μM nocodazole (a microtubule- depolymerizing agent) for 24 hours. Then, nocodazole was removed, a portion of the cells were collected and fixed, and the other portion was cultured for 24 and 48 hours in nocodazole-free medium. Subsequently, the cells were harvested and fixed in fixed in 70% ethanol for 60 min at minus 20°C, treated with RNase (100 μg/mL) for 30 min at 37°C, and stained with propidium iodide (50μg/mL). After that, the cells were analyzed in FACSCalibur flow cytometry (BD Biosciences), and cell cycle distribution was determinated using ModFit LT V3-.3.11 software.

**Immunohistochemistry**

Tumor fragments were fixed in 10% neutral-buffered [formalin](https://www.sciencedirect.com/topics/medicine-and-dentistry/formaldehyde), embedded in paraffin, and sectioned for histopathologic and immunohistochemical analyses. After deparaffinization, rehydration, and heat-induced epitope retrieval in citrate buffer (pH 6.0), sections were incubated with antibodies against Ki67 (M7240, Dako), CD44 (#3570, Cell Signaling Technology), PCK26 (ab6401), E-cadherin (#3195, Cell Signaling Technology) and p53 (sc-126, Santa Cruz Biotechnology). Tumor sections were counterstained with Harry´s hematoxylin. Images were acquired at ×20 magnification using an Aperio ScanScope Scanner (Aperio Technologies).

**Supplementary Figures**

**
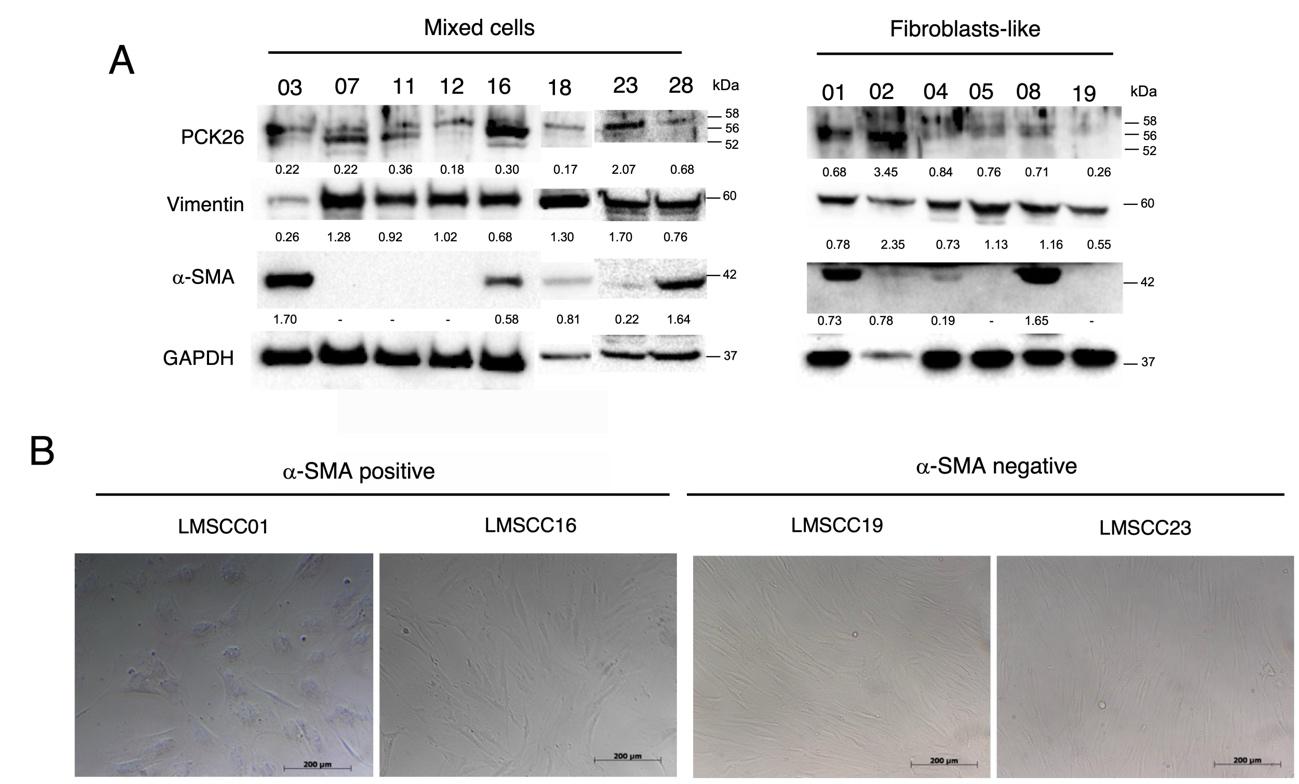
**

**Supplementary Figure 1. A.** Protein analysis of pan- cytokeratin, vimentin, and alpha-SMA expression in mixed-cells (fibroblasts and epithelial cells) and in fibroblast-like cells (>90% of fibroblasts-like cells). GAPDH was used as a loading control and the expression calculated using ImageJ software. **B.** Representative images showing the morphology of alpha-SMA-positive and alpha-SMA-negative cells. Images were captured using a 20x objective; scale bar: 200μm.


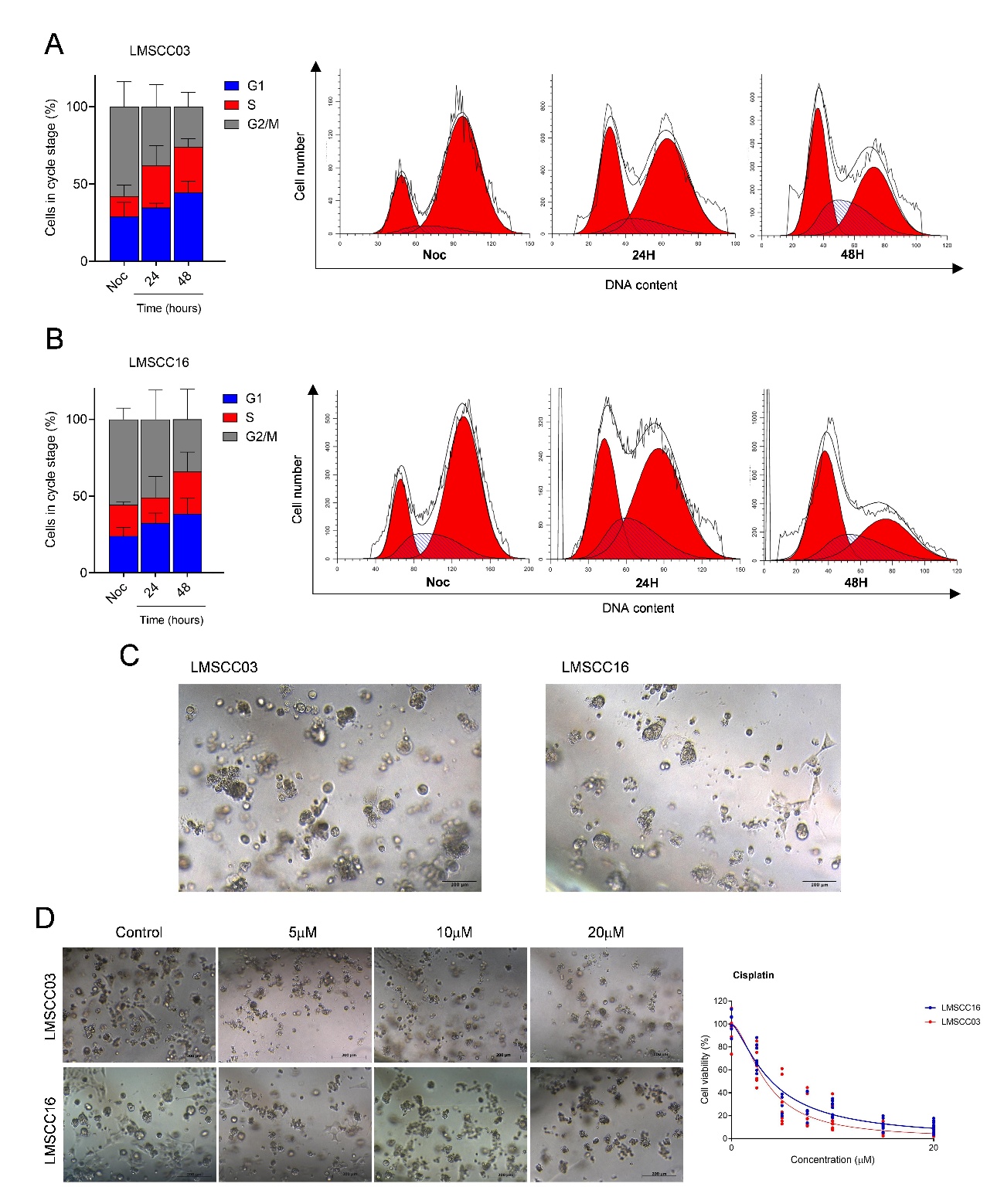


**Supplementary Figure 2.** The proportion of cells in G1 phase, S phase, G2/M phase to the total number of cells of LMSCC03 **(A)** and LMSCC16 **(B)**. Representative phase-contrast images showing the morphology of alpha-SMA-positive and alpha-SMA-negative fibroblasts. Images were captured using a 20×objective scale bar: 200μm **(C)**. Brightfield images and graphs of organoids from LMSCC cells treated with different Cisplatin concentrations (5, 10, 20μM) for 72hours. Scale bars 200μm **(D)**.


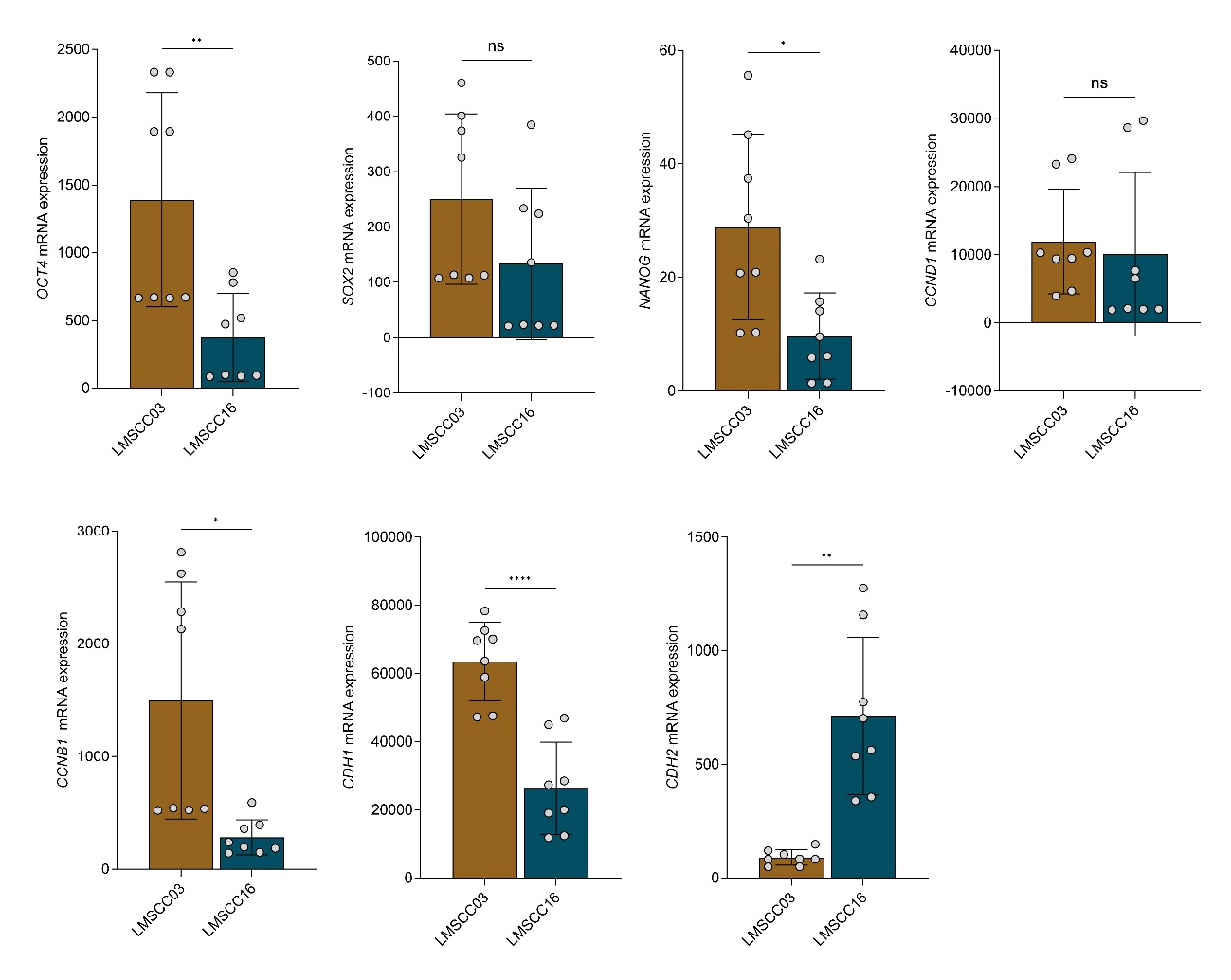


**Supplementary Figure 3.** Relative mRNA expression of pluripotency markers (*OCT4*, *SOX2*, *NANOG*), cell cycle regulators (*CCND1*, *CCNB1*), and epithelial–mesenchymal transition markers (*CDH1* and *CDH2*) in LMSCC03- and LMSCC16-derived spheroids. Statistical comparisons between LMSCC03 and LMSCC16 spheroids were performed using an unpaired Student’s t-test. Significance levels were defined as ns > 0.05; *p<0.05; **p<0.01; ***p<0.001; ****p<0.0001.

***Supplementary Uncropped Western Blots***

Uncropped western blot images are provided in Supplementary Figures 4–8 below.

**Supplementary Figure 4-** Original blot corresponding Figure 2D (LMSCC03 cell line).

**
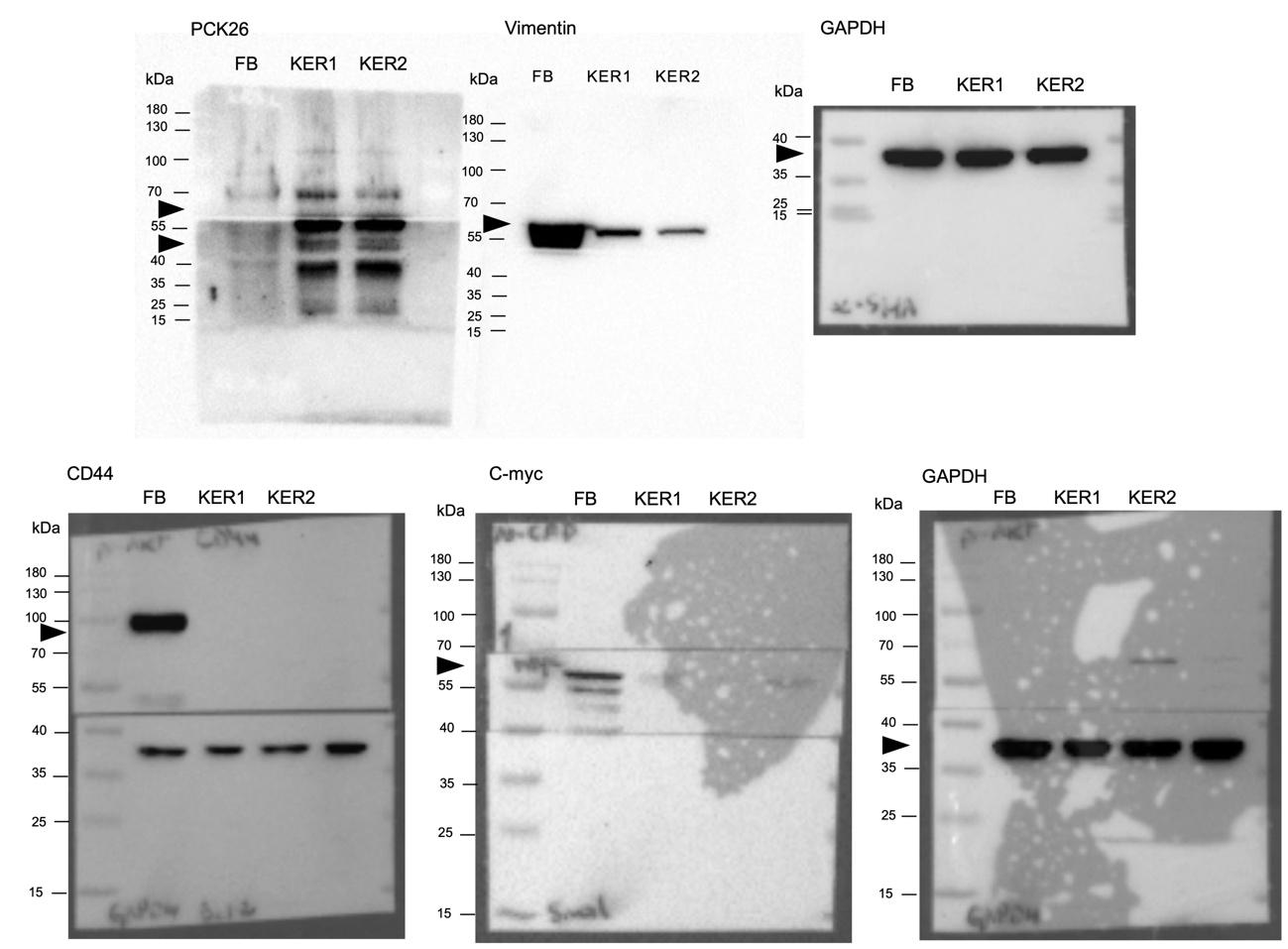
**

Full-length western blots for the detection of PCK26, vimentin, GAPDH, CD44, and C-myc. Molecular weight markers (kDa) are indicated on the left. The cropped regions used in the Figure 2D are shown using arrows.

**Supplementary Figure 5-** Original blot corresponding Figure 2D (LMSCC16 cell line)

**
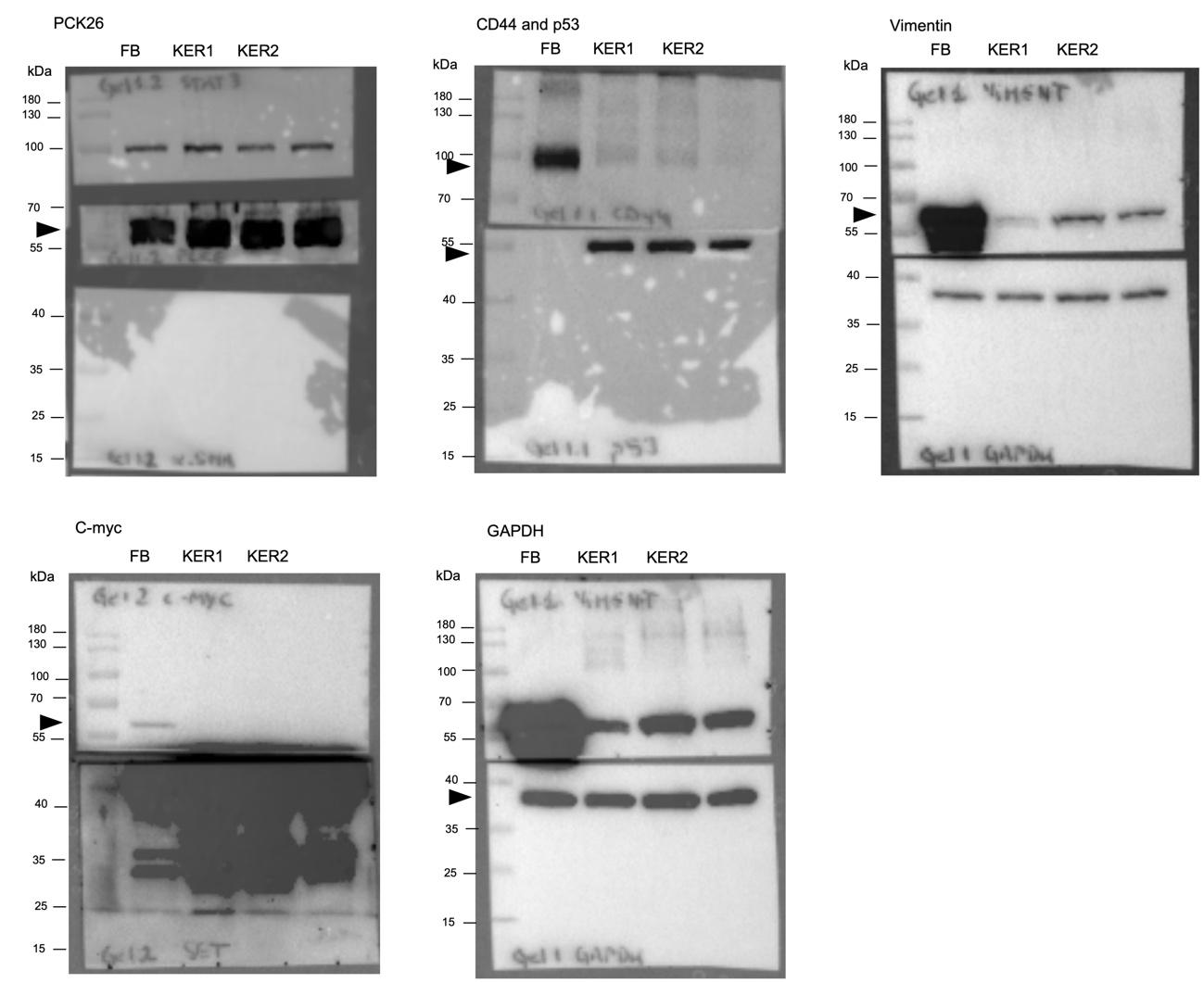
**

Full-length western blots for the detection of PCK26, CD44, p53, vimentin, C-myc, and GAPDH. Molecular weight markers (kDa) are indicated on the left. The cropped regions used in Figure 2D are shown using arrows.

**Figure Supplementary 6.** Original blot corresponding Figure S1A (Mixed cells)

**
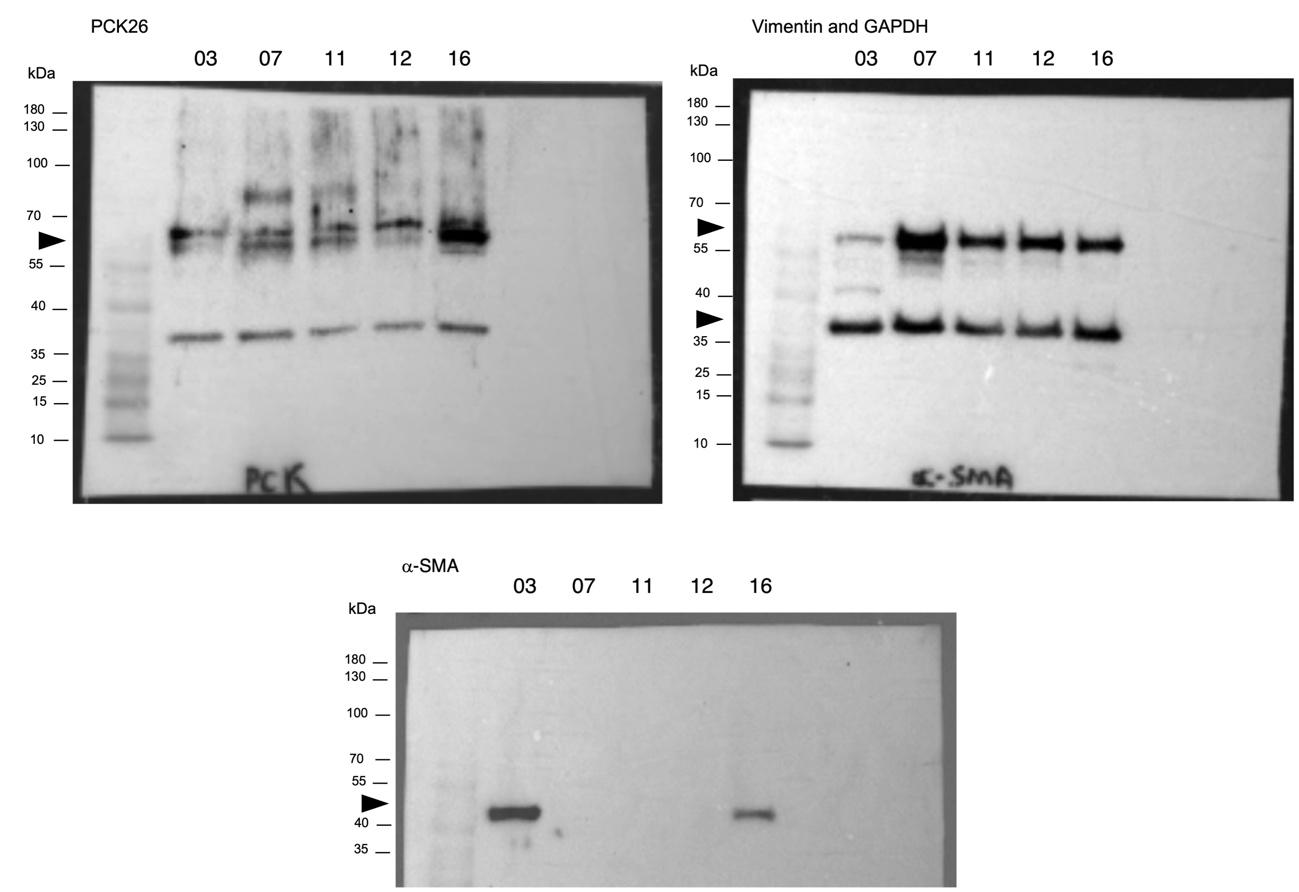
**

Full-length western blots for the detection of PCK26, vimentin, GAPDH, and alpha-SMA. Molecular weight markers (kDa) are indicated on the left. The cropped regions used in the Figure S1A are shown using arrows.

**Figure Supplementary 7.** Original blot corresponding Figure S1A (Fibroblasts-like cells)

**
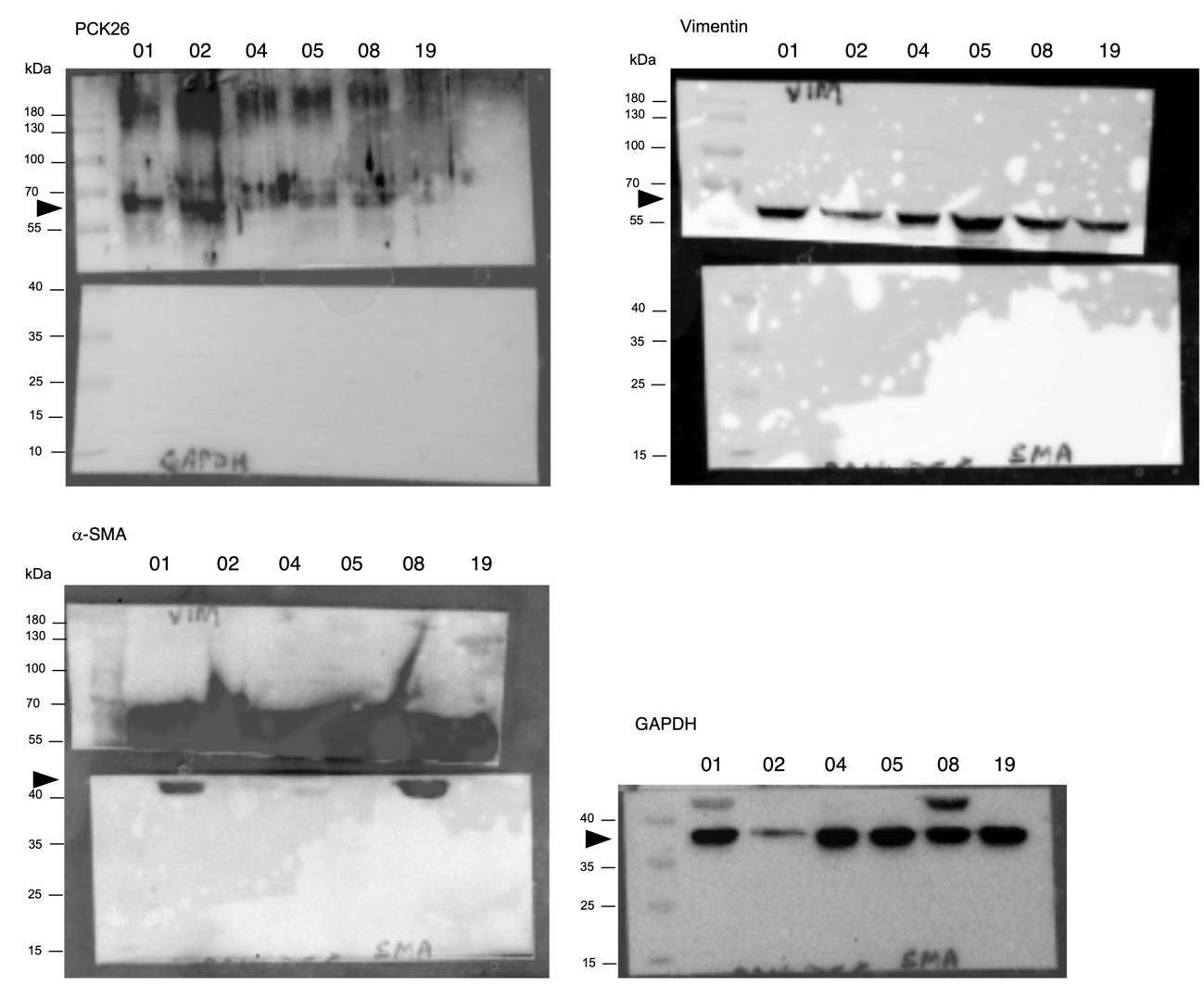
**

Full-length western blots for the detection of PCK26, vimentin, alpha-SMA, and GAPDH. Molecular weight markers (kDa) are indicated on the left. The cropped regions used in the Figure S1A are shown using arrows.

**Figure Supplementary 8.** Original blot corresponding Figure S1A (Fibroblasts-like cells)

**
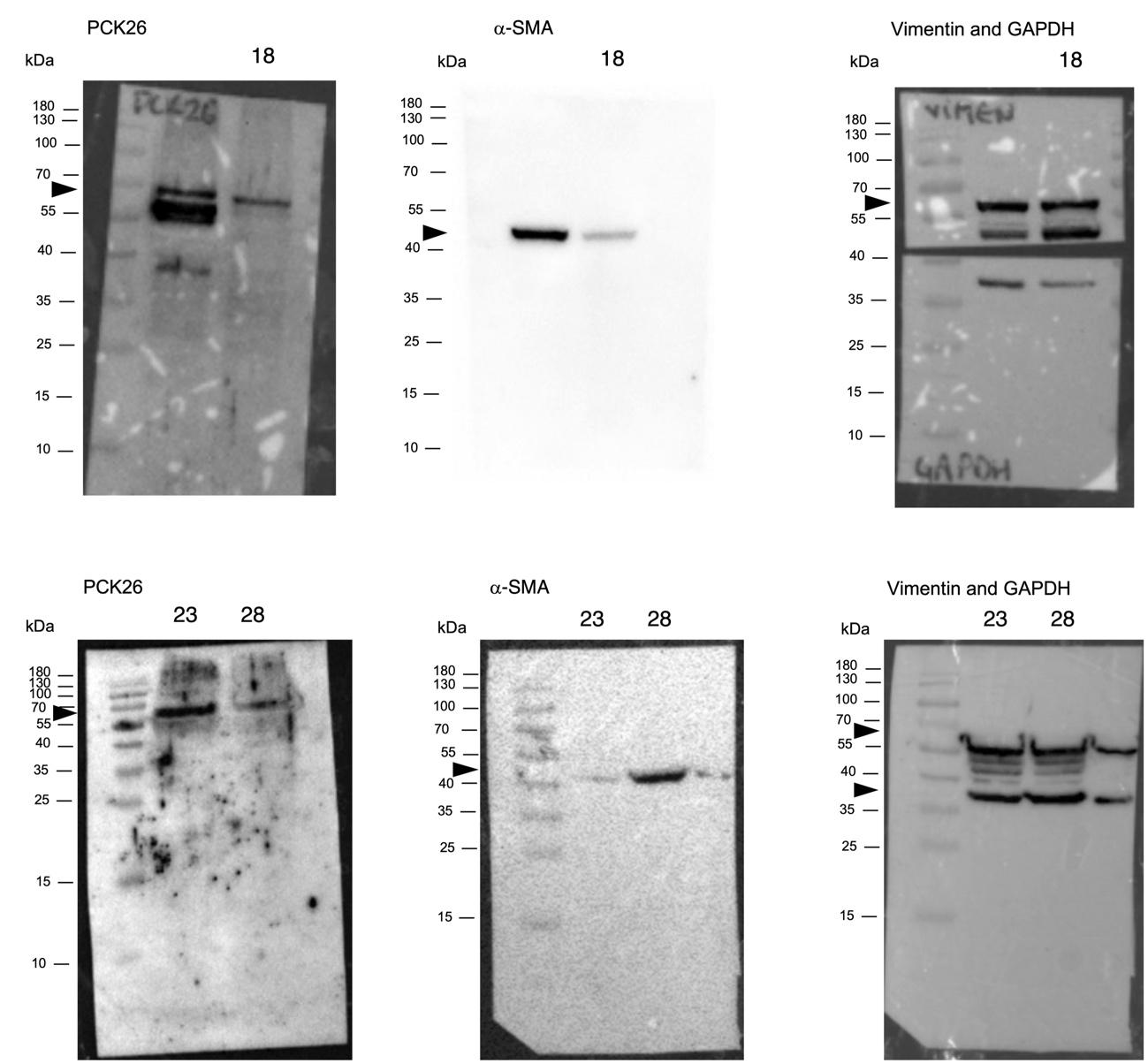
**

Full-length western blots for the detection of PCK26, alpha-SMA, vimentin, and GAPDH. Molecular weight markers (kDa) are indicated on the left. The cropped regions used in the Figure S1A are shown using arrows.
