# Supplementary material for: Novel patient-derived tongue squamous cell carcinoma cell lines from non-smokers: 3D and in vivo models for drug response studies
Source: Med Oncol. 2026 Jun 29;43(8):206. doi: 10.1007/s12032-026-03311-9 (PMC13314703; doi:10.1007/s12032-026-03311-9)
Supplement: Supplementary file 2 — Supplementary Material 2 [file 12032_2026_3311_MOESM2_ESM.docx]

**Supplementary Table 1.** List of established TSCC cell lines and their associated molecular and clinical features.

| Cell line name | Clinical characteristics | Molecular alterations | Smoke status | References |
| --- | --- | --- | --- | --- |
| AMC-HN-1 | Male, 54Y | - | Not found | [1] |
| AMC-HN-4 | Female, 65Y | - | Not found | [1] |
| AW 8507 | Female, 50Y | - | Not found | [2] |
| BICR-16 | Male, - | *TP53* mutation (c.438G>A) | Not found | [3] |
| BICR-22 | Male, 88Y, derived of lymph node metastasis | *TP53* mutation (c.920-1G>T) | Not found | [3] |
| BICR-31 | Male, - | *TP53* mutation (c.518_520delTGA) | Not found | [3] |
| BICR-56 | Male, - | *TP53* mutation (c.376-1G>A) | Not found | [3] |
| Cal27 | Male, 56Y | *TERT* mutation (c.228C>T)  *TP53* mutation (c.578A>T) | Not found | [4,5] |
| Cal33 | Male, 69Y | *TMPRSS2* mutation (c.23G>T)  *TP53* mutation (c.524G>A) | Not found | [4] |
| CCH-FAHNSCC-1 | Male, - | - | Not found | [6] |
| SCC-4 | Male, 55Y | - | Not found | [7] |
| SCC-15 | Male, 55Y | - | Not found | [7] |
| SCC-25 | Male, 70Y | *TP53* mutation (c.626_627delGA) | Not found | [7] |
| SCC-9 | Male, 25Y | *TP53* mutation (c.822_853del) | Not found | [7] |
| UM-SCC-4 | Female, 48Y | *TP53* mutation (c.378C>G, c.637C>T) | Yes | [8] |
| UM-SCC-47 | Male, 53Y | *NOTCH1* mutation (c.574G>T) | Not found | [8] |
| UPCI-SCC-040 | Male, 50Y | *TP53* wildtype | No | [9] |
| UPCI-SCC-062 | Female, 18Y | *TP53* wildtype | Yes | [9] |
| UPCI-SCC-068 | Male, 60Y | *TP53* wildtype | Yes | [9] |
| UPCI-SCC-075 | Male, 67Y | *TP53* wildtype | Yes | [9] |
| UPCI-SCC-090 | Female, 27Y | HPV positive, TP53 wildtype | Yes | [9] |
| UPCI-SCC-103 | Female, 27Y | *TP53* mutation (c.916C>T) | Yes | [9] |
| UPCI:SCC152 | Male, 47Y | HPV positive, *TP53* wildtype | Yes | [9] |
| UPCI:SCC154 | Male, 54Y | HPV positive, *TP53* wildtype | Yes | [9] |
| UPSF:OT-1109 | Female, 44Y | *TP53* mutation (c.743G>A) | No | [10] |
| WSU-HN4 | Female, - | - | Not found | [11] |
| WSU-HN12  (same patient WSU-HN4) | Female, -  derived of lymph node metastasis | *TP53* mutation (c.672+1G>A) | Not found | [11,12] |
| WSU-HN13 | Female, - | *TP53* mutation (c.517G>T) | Not found | [11] |
| WSU-HN6 | Male, - | *TP53* mutation (c.536A>T) | Not found | [11] |
| LK0412 | Female, 50Y | *TP53* mutation (c.734G>A) | Not found | [13] |
| OC1 | Male, 42Y | - | Yes | [14] |
| OC2 | Male, 51Y | HPV positive | Yes | [14] |

**References**

1. Kim SY, Chu KC, Lee HR, Lee KS, Carey TE. Establishment and characterization of nine new head and neck cancer cell lines. Acta Otolaryngol. 1997;117:775–84. https://doi.org/10.3109/00016489709113477

2. Tatake RJ, Rajaram N, Damle RN, Balsara B, Bhisey AN, Gangal SG. Establishment and characterization of four new squamous cell carcinoma cell lines derived from oral tumors. J Cancer Res Clin Oncol. 1990;116:179–86. https://doi.org/10.1007/BF01612674

3. Burns JE, Baird MC, Clark LJ, Burns PA, Edington K, Chapman C, et al. Gene mutations and increased levels of p53 protein in human squamous cell carcinomas and their cell lines. Br J Cancer. 1993;67:1274–84. https://doi.org/10.1038/bjc.1993.238

4. Gioanni J, Fischel JL, Lambert JC, Demard F, Mazeau C, Zanghellini E, et al. Two new human tumor cell lines derived from squamous cell carcinomas of the tongue: establishment, characterization and response to cytotoxic treatment. Eur J Cancer Clin Oncol. 1988;24:1445–55. https://doi.org/10.1016/0277-5379(88)90335-5

5. Martin D, Abba MC, Molinolo AA, Vitale-Cross L, Wang Z, Zaida M, et al. The head and neck cancer cell oncogenome: a platform for the development of precision molecular therapies. Oncotarget. 2014;5:8906–23. https://doi.org/10.18632/oncotarget.2417

6. Nguyen HT, Tang W, Webster ALH, Whiteaker JR, Chandler CM, Errazquin R, et al. Fanconi anemia-isogenic head and neck cancer cell line pairs: A basic and translational science resource. Int J Cancer. 2023;153:183–96. https://doi.org/10.1002/ijc.34506

7. Rheinwald JG, Beckett MA. Tumorigenic keratinocyte lines requiring anchorage and fibroblast support cultured from human squamous cell carcinomas. Cancer Res. 1981;41:1657–63.

8. Bradford CR, Zacks SE, Androphy EJ, Gregoire L, Lancaster WD, Carey TE. Human papillomavirus DNA sequences in cell lines derived from head and neck squamous cell carcinomas. Otolaryngol Head Neck Surg. 1991;104:303–10. https://doi.org/10.1177/019459989110400304

9. White JS, Weissfeld JL, Ragin CCR, Rossie KM, Martin CL, Shuster M, et al. The influence of clinical and demographic risk factors on the establishment of head and neck squamous cell carcinoma cell lines. Oral Oncol. 2007;43:701–12. https://doi.org/10.1016/j.oraloncology.2006.09.001

10. Wang SJ, Asthana S, van Zante A, Heaton CM, Phuchareon J, Stein L, et al. Establishment and characterization of an oral tongue squamous cell carcinoma cell line from a never-smoking patient. Oral Oncol. 2017;69:1–10. https://doi.org/10.1016/j.oraloncology.2017.03.020

11. Cardinali M, Pietraszkiewicz H, Ensley JF, Robbins KC. Tyrosine phosphorylation as a marker for aberrantly regulated growth-promoting pathways in cell lines derived from head and neck malignancies. Int J Cancer. 1995;61:98–103. https://doi.org/10.1002/ijc.2910610117

12. Yeudall WA, Jakus J, Ensley JF, Robbins KC. Functional characterization of p53 molecules expressed in human squamous cell carcinomas of the head and neck. Mol Carcinog. 1997;18:89–96. https://doi.org/10.1002/(sici)1098-2744(199702)18:2%3C89::aid-mc4%3E3.0.co;2-l

13. Roberg K, Ceder R, Farnebo L, Norberg-Spaak L, Grafström RC. Multiple genotypic aberrances associate to terminal differentiation-deficiency of an oral squamous cell carcinoma in serum-free culture. Differentiation. 2008;76:868–80. https://doi.org/10.1111/j.1432-0436.2008.00267.x

14. Wong DY, Chang KW, Chen CF, Chang RC. Characterization of two new cell lines derived from oral cavity human squamous cell carcinomas--OC1 and OC2. J Oral Maxillofac Surg. 1990;48:385–90. https://doi.org/10.1016/0278-2391(90)90436-6
