# Supplementary material for: Novel patient-derived tongue squamous cell carcinoma cell lines from non-smokers: 3D and in vivo models for drug response studies
Source: Med Oncol. 2026 Jun 29;43(8):206. doi: 10.1007/s12032-026-03311-9 (PMC13314703; doi:10.1007/s12032-026-03311-9)
Supplement: Supplementary file 3 — Supplementary Material 3 [file 12032_2026_3311_MOESM3_ESM.docx]

**Supplementary Table 2***.* ﻿Expression of Pan Cytokeratin, α-SMA, and Vimentin in primary cell culture derived of OSCC patients.

| **Patients** | **Pathologic stage (TNM)** | **Pan Cytokeratin** | **α-SMA** | **Vimentin** |
| --- | --- | --- | --- | --- |
| LMSCC01 | pT2, pN2b, Mx | High-intensity | Positive | Low-intensity |
| LMSCC02 | pT4a | High-intensity | Positive | High-intensity |
| LMSCC03 | pT3,pN3b | Low-intensity | Positive | Low-intensity |
| LMSCC04 | pT4a, pN3b | High-intensity | Positive | Low-intensity |
| LMSCC05 | pT3, pN2c | High-intensity | Negative | High-intensity |
| LMSCC07 | pT4a, pN0 | Low-intensity | Negative | High-intensity |
| LMSCC08 | pT4a, NA | High-intensity | Positive | High-intensity |
| LMSCC11 | pT2, pN3b | Low-intensity | Negative | High-intensity |
| LMSCC12 | pT4a, pN3b | Low-intensity | Negative | High-intensity |
| LMSCC16 | pT3, pN3b | Low-intensity | Positive | Low-intensity |
| LMSCC18 | pT2, pN0 | Low-intensity | Positive | High-intensity |
| LMSCC19 | pT3, pN3b | Low-intensity | Negative | Low-intensity |
| LMSCC23 | pT4a, pN2b, M0 | High-intensity | Positive | High-intensity |
| LMSCC28 | pT4a, pN3b | High-intensity | Positive | Low-intensity |

TNM- classification is a system for classifying a malignancy using T (tumor), N (nodes), and M (metastasis). pT- size and extent of the primary tumor, pN- involvement of regional lymph nodes, M- presence of distant metastasis.
