# Supplementary material for: Novel patient-derived tongue squamous cell carcinoma cell lines from non-smokers: 3D and in vivo models for drug response studies
Source: Med Oncol. 2026 Jun 29;43(8):206. doi: 10.1007/s12032-026-03311-9 (PMC13314703; doi:10.1007/s12032-026-03311-9)
Supplement: Supplementary file 4 — Supplementary Material 4 [file 12032_2026_3311_MOESM4_ESM.docx]

**Supplementary Table 3**. Short tandem repeat (STR) allelic profile of LMSCC03 and LMSCC16.

| *Locus* | LMSCC03 | LMSCC16 |
| --- | --- | --- |
| CSF1PO | 11 | 12 |
| D13S317 | 8,12 | 10 |
| D16S539 | 11,12 | 13 |
| D5S818 | 11 | 13 |
| D7S820 | 9 | 12 |
| THO | 9,9.3 | 6,9 |
| TPOX | 8 | 8,9 |
| vWA | 18 | 16,18 |
