# Supplementary material for: Novel patient-derived tongue squamous cell carcinoma cell lines from non-smokers: 3D and in vivo models for drug response studies
Source: Med Oncol. 2026 Jun 29;43(8):206. doi: 10.1007/s12032-026-03311-9 (PMC13314703; doi:10.1007/s12032-026-03311-9)
Supplement: Supplementary file 5 — Supplementary Material 5 [file 12032_2026_3311_MOESM5_ESM.docx]

**Supplementary Table 4**. Primers sequence used for qRT-PCR.

| Target gene | Primer sequence Foward (5’-3’) | Primer sequence Reverse (5’-3’) |
| --- | --- | --- |
| *OCT4* | GTCCGAGTGTGGTTCTGTA | CTCAGTTTGAATGCATGGGA |
| *SOX2* | GCTACAGCATGATGCAGGACCA | TCTGCGAGCTGGTCATGGAGTT |
| *NANOG* | CTCCAACATCCTGAACCTCAGC | CGTCACACCATTGCTATTCTTCG |
| *CCND1* | GTTCATTTCCAATCCGCCT | GCGGTAGTAGGACAGGAAGT |
| *CCNB1* | TGGATGCAGAAGATGGAGCT | TCTGACTGCTTGCTCTCCT |
| *CDH1* | ATTCCTGCCATTCTGGGGAT | GCAGTAAGGGCTCTTTGACC |
| *CDH2* | TAAAGCGGCTGACAATGACC | TTAAGGGACCTCAAGGACCC |
| *RPL27* | ACAATCACCTAATGCCCACA | GCCTGTCTTGTACTCTCTTCAA |
